# Low-Temperature Stress Impairs Reproductive Performance and Olfactory Behaviors in *Tuta absoluta* via Metabolic and Transcriptional Changes

**DOI:** 10.3390/insects17070706

**Published:** 2026-07-07

**Authors:** Bo Feng, Chuanhong Feng, Zhigang Yang, Genyun Liang, Liping Xiong, Xi Yang, Jiatao Huang, Tao Hu, Lingzhi Huang, Yong Yin, Kaidi Zheng

**Affiliations:** 1 Mianyang Teachers’ College, College of Biology and Pharmaceutical Sciences, Mianyang 621000, China; fb825028@aliyun.com (B.F.); x2016766@163.com (L.X.); 15282618441@163.com (X.Y.); 15700210683@163.com (J.H.); 2 Plant Protection Station of Sichuan Provincial Department of Agriculture and Rural Affairs, Chengdu 610041, China; fengchuanhong8@163.com (C.F.); cqhutaozjjt@163.com (T.H.); huanglingzhi0421@163.com (L.H.); 3 Leshan Plant Protection and Plant Quarantine Station, Leshan 614000, China; leshanshipps@163.com; 4 Horticultural Research Institute, Sichuan Academy of Agricultural Sciences, Chengdu 610066, China; liang_gyrb@scsaas.cn

**Keywords:** low-temperature stress, *Tuta absoluta*, life traits, energy metabolism, antioxidant defense, metabolomics, transcriptomics, climate change

## Abstract

Low temperature critically pressures insect pest distributions and population dynamics; however, integrated understanding of cold stress responses in invasive pests, particularly at the adult stage, remains limited. Here, we investigated the integrated physiological, biochemical, metabolomic and transcriptomic responses of *Tuta absoluta* (Meyrick, 1917) exposed to control (CT: 25 °C), moderate cold stress (MCS: 15 °C) and severe cold stress (SCS: 5 °C) conditions. Our results revealed that low-temperature stress impaired *T. absoluta* emergence, survival, fecundity and egg hatching in a stress intensity-dependent manner. Also, low temperature reduced the olfactory-mediated host-seeking behaviors of *T. absoluta* females. The biochemical analyses revealed depletion of triglycerides, glycogen and Na^+^/K^+^-ATPase activity alongside compensatory trehalose accumulations, while antioxidant enzyme activities (SOD, POD, CAT) were differentially modulated indicating progressive oxidative defense impairment under low temperature. Untargeted metabolomic profiling identified extensive differential metabolite accumulations, revealing systematic dysregulation of alkaloid biosynthesis, amino acid metabolism and central carbon metabolic pathways. Transcriptomic analysis identified several differentially expressed genes (DEGs) enriched in thermogenesis, oxidative phosphorylation, MAPK signaling and neurodegeneration-associated pathways. Furthermore, integrative transcriptome–metabolome analysis revealed coordinated gene–metabolite regulatory networks scaling systematically with cold stress intensity. These findings advance our mechanistic understanding of thermal stress adaptations in *T. absoluta* and may contribute to the development of climate-informed pest management strategies.

## Simple Summary

The tomato leaf miner, *Tuta absoluta*, is a devastating global pest that thrives in warm climates. As temperatures shift due to climate change, understanding how low temperature affects this pest’s survival and reproduction is critical for predicting its geographic spread. We exposed adult tomato leaf miners to different low-temperature conditions (25 °C, 15 °C and 5 °C) under controlled laboratory conditions to simulate optimal, moderate and severe cold stress exposure and found that even moderately cool temperatures dramatically reduced their egg production, larval survival and ability to locate host plants. Using omics techniques, we analyzed metabolic and genetic changes in the *T. absoluta* and discovered that low temperature disrupts their energy metabolism, depletes their nutrient reserves and damages cellular protection systems. These molecular changes may help explain why low temperatures prevent the insects from reproducing and finding food. Our findings suggest that cooler conditions may impose physiological and behavioral constraints on this pest, providing mechanistic insights that could help inform future studies on climate-related population dynamics and pest management strategies.

## 1. Introduction

Among invasive lepidopteran pests, the tomato leaf miner, *Tuta absoluta* (Meyrick, 1917), represents a major global threat to solanaceous crops, particularly tomato production [1,2]. Following its rapid expansions from South America into Europe, Africa and Asia, these species have demonstrated exceptional ecological flexibility, enabling successful establishment across diverse climatic regions [3,4,5]. While its invasion success has traditionally been attributed to high reproductive potential and concealed feeding behavior, increasing evidence suggests that physiological adaptability plays a central role in its persistence under varying environmental conditions. In particular, the ability of *T. absoluta* to withstand temperature fluctuations is a critical determinant of its geographic spread and population stability, especially in regions experiencing seasonal cold stress [3,6,7]. Temperature is one of the most influential environmental factors regulating insect physiology, behavior and distribution. For a thermophilic species such as *T. absoluta*, favorable temperatures enhance reproductive output and dispersal capacity, whereas low temperatures can impose significant physiological constraints [8]. As this pest continues to expand into temperate zones under shifting climate regimes, populations are increasingly exposed to fluctuating thermal environments, including optimal developmental temperatures (~20–30 °C) and episodic cold conditions that may drop to 10–15 °C during seasonal transitions, and in extreme cases approach near-freezing temperatures during short-term cold spells. These fluctuations can impose strong constraints on survival, dispersal and reproduction. Therefore, a key question emerges: how do adult moths respond to suboptimal and cold-temperature conditions that directly affect survival, dispersal and reproduction [9,10,11]? Addressing this concern is important for improving predictions of invasion dynamics, understanding seasonal population fluctuations and identifying potential vulnerabilities that could be exploited for pest management.

Exposure to low temperatures can disrupt cellular homeostasis and impose energetic constraints, requiring insects to adjust internal resources to maintain essential physiological functions [12]. In adult insects, these constraints are particularly critical, because energy demand is strongly associated with flight activity, reproductive output and host-seeking behaviors [13]. Low temperature can alter metabolic pathways by shifting the balance between energy storage and utilization, leading to depletion of key reserves such as lipids and carbohydrates, while also modifying enzymatic activities involved in energy turnover [14,15]. At the same time, low temperature impairs mitochondrial function and electron transport processes, resulting in excessive accumulation of reactive oxygen species (ROS) and increased oxidative stress [13,16]. In response, insects activate antioxidant defense systems to maintain redox balance and protect cellular integrity, highlighting the tight coupling between energy metabolism and oxidative balance, which in turn will negatively affect key ecological traits such as reproduction and host-seeking behavior [17]. In natural environments, insect populations are exposed to dynamic thermal regimes that include both heat waves and periodic cold stress depending on season and geography [18]. While high-temperature stress has been widely studied for its impacts on protein stability, metabolism and survival, low-temperature stress remains comparatively underexplored despite its strong role in limiting overwintering success and seasonal population establishment. For thermophilic invasive pests such as *Tuta absoluta*, episodic cold exposure in newly colonized temperate regions can strongly constrain energy metabolism, neuromuscular function and reproduction [11]. Therefore, examining low-temperature responses is essential to complement existing knowledge of heat stress and to better predict thermal limits and climate-driven range expansion.

Recent advances in systems biology have demonstrated that insect responses to environmental stresses are governed by coordinated molecular and metabolic reprogramming rather than isolated physiological changes [14,19]. High-throughput omics approaches have revealed that temperature stress can induce widespread alterations in gene expression and metabolite profiles, affecting pathways related to energy metabolism, stress resistance and cellular maintenance [14,20]. The integration of transcriptomic and metabolomic analyses has proven particularly effective for uncovering the mechanistic basis of stress adaptation, providing insights into how molecular regulation translates into physiological outcomes [21]. However, such integrative investigations remain limited for many invasive pest species, including *T. absoluta*, particularly at the adult stage where behavior and ecological performance are directly expressed.

Compared with immature stages, adult moths play a decisive role in determining population expansion through dispersal, host location and reproduction [22,23]. These processes depend heavily on the functionality of flight muscles, sensory systems and reproductive physiology, all of which are highly sensitive to temperature variations [24,25]. Despite this, most previous studies on *T. absoluta* have focused on developmental thresholds, larval performance, or control strategies, while the physiological and molecular responses of adults to low temperature remain poorly characterized [26,27]. In particular, the effects of cold exposure on multiple interconnected processes including energy metabolism, oxidative balance and behavior, such as olfactory-driven host selection and oviposition, have not been systematically explored.

The objectives of the present study are to investigate how low temperature influences physiological performance, metabolic regulation and behavioral responses in *T. absoluta* adults. By integrating measurements of energy metabolism and antioxidant defense with metabolomic and transcriptomic profiling, we aim to elucidate the coordinated mechanisms underlying temperature-induced stress responses. We hypothesize that low temperature induces a graded, temperature-dependent response in adult moths, characterized by depletion of energy reserves, disruption of oxidative balance and coordinated molecular reprogramming, ultimately reducing olfactory behavior, reproduction and ecological fitness.

## 2. Materials and Methods

### 2.1. Plant–Insect Material and Experimental Conditions

The tomato leaf miner, *Tuta absoluta* (Meyrick, 1917), was maintained under laboratory conditions for more than 20 generations to ensure a stable and uniform population. Insects were reared on tomato plants in a controlled environment at 25 ± 1 °C, with a 16:8 h light: dark photoperiod and 70% relative humidity. Tomato plants (*Solanum lycopersicum*, *cv*. Heinz) were established from surface-sterilized seeds. Briefly, seeds were disinfected in 1% sodium hypochlorite (NaClO) for 5 min and rinsed three times with sterile distilled water. Seeds were then germinated on moist filter paper in Petri dishes (8.5 cm diameter). After germination, seedlings (2–3 cm height) were transplanted into plastic pots (115 × 110 mm) containing a sterilized peat-based substrate (Shenzhen Shenglvyuan Horticulture Co., Ltd., Shenzhen, China) [28]. Plants were grown in a greenhouse under controlled conditions (25 ± 1 °C, 16:8 h light: dark photoperiod, light intensity of 600–800 μmol m^−2^ s^−1^) and watered daily. For experimental assays, newly emerged *T. absoluta* adults were collected from the stock culture to ensure uniform age. Adults were exposed to three temperature regimes: control stress (CT, 25 °C), moderate cold stress (MCS, 15 °C) and severe cold stress (SCS, 5 °C), using separate climate-controlled incubators (MLR-352-PE, Panasonic, Tokyo, Japan) [29], where temperatures were continuously monitored using an independent calibrated digital thermometer placed inside the incubator to verify real-time environmental conditions. All treatments were conducted under identical photoperiod (16:8 h light: dark) and light intensity (400 μmol m^−2^ s^−1^) conditions. *T. absoluta* adults maintained under temperature treatments (CT, MCS and SCS) for 7 days were used for biochemical, metabolomic and transcriptomic studies.

### 2.2. Leaf Minor Life History Traits Under Temperature Treatments

To determine *T. absoluta* adult emergence, pupae were collected from the stock culture; because pupation occurs within leaf tissues, infested-leaf sections containing intact pupae were carefully excised using a sterile blade without disturbing the pupal chamber. The pupae were placed on moist cotton (10 pupae/replicate) within glass tubes (50 mL) and maintained under the three temperature treatments (CT, MCS and SCS) with a photoperiod of 16:8 h light: dark and relative humidity of 70%. Adult emergence was recorded daily until no further emergence occurred and emergence rate was calculated as the percentage (%) of emerged adults relative to the total number of pupae and the experiment was repeated three times.

To evaluate survival rate, newly emerged adults of uniform age were collected from the stock colony and transferred into glass tubes (50 mL: (10 adults/replicate) to prevent interaction effects. Each individual was provided with a 10% honey solution as a food source. The tubes were maintained under the three temperature treatments (CT, MCS and SCS) under identical photoperiod conditions. Survival rate was recorded and the experiment was repeated three times.

For oviposition assays, *T. absoluta* pairs were first maintained under control conditions (25 °C) for 3 days to ensure successful mating. After mating, individuals were transferred into separate 500 mL plastic containers covered with muslin cloth and assigned to one of three temperature treatments (CT: 25 °C, MCS: 15 °C and SCS: 5 °C) for oviposition assessment. Adults were provided with a tomato leaf for egg laying and a 10% honey solution as a food source. The number of eggs laid per female was recorded daily under each temperature treatment and the experiment was repeated three times. For egg hatching assays, eggs laid on the same day under controlled rearing conditions at 25 °C were collected to ensure uniform developmental age and were then distributed evenly among the three temperature treatments (CT, MCS and SCS). Hatching success was monitored daily until no further hatching occurred and calculated as the percentage (%) of hatched larvae relative to the total number of eggs and the experiment was repeated three times.

### 2.3. Biochemical Analysis of Energy Metabolism

Glycogen and trehalose contents were measured using commercial assay kits (Nanjing Jiancheng Bioengineering Institute, Beijing, China) according to the manufacturer’s protocols. Glycogen contents (Cat. No. A043-1-1) were measured spectrophotometrically at 620 nm following acid-hydrolysis and anthrone reactions. Trehalose content (Cat. No. A149-1-1) was measured from adults following extraction and centrifugation at 8000 rpm for 10 min, followed by reaction with assay reagents and brief heating and quantification by measuring absorbances at 620 nm. The levels of triglycerides (Cat. No. BC0625, Solarbio, Beijing, China) and ATPase (Cat. No. BC0065, Solarbio, Beijing, China) were measured following the manufacturer’s instructions. Each biological replicate consisted of 10 adult individuals per temperature treatment, with five independent biological replicates per assay.

### 2.4. Antioxidant Enzyme Activity Assay

The antioxidant enzyme activities were quantified using commercially available assay kits (Solarbio Life Sciences, Beijing, China) following the manufacturer’s protocols with slight modifications. Briefly, *T. absoluta* adults were homogenized in ice-cold 0.05 M phosphate buffer (pH 7.8) supplemented with 0.1 mM EDTA and 1% (*w*/*v*) polyvinylpyrrolidone to stabilize enzyme activities and minimize oxidative interferences. Homogenization was performed using a FastPrep^®^-24 Classic instrument (Pudong, Shanghai, China), after which the homogenates were centrifuged at 11,000 rpm for 10 min at 4 °C. The resulting supernatants were carefully collected and stored at −70 °C until further analysis [30]. Peroxidase (POD) activity was measured based on hydrogen peroxide (H_2_O_2_)-dependent oxidation of the substrate with changes in absorbance monitored at 420 nm, superoxide dismutase (SOD) activity was measured using a xanthine–xanthine oxidase system in which the inhibition of superoxide radical generation was quantified spectrophotometrically at 550 nm and catalase (CAT) activity was assessed by monitoring the decomposition of H_2_O_2_ and the subsequent formation of a stable complex with ammonium molybdate with absorbance recorded at 405 nm. All enzyme activities were normalized to protein concentrations determined using the Bradford assay (Coomassie Brilliant Blue G-250 method) and each treatment was conducted with 5 independent biological replicates (10 adult individuals per replicate).

### 2.5. Olfactory Choice Assays

Olfactory responses of adult female *T. absoluta* to host plant volatiles were evaluated using a glass Y-tube olfactometer (16 cm stem, two 10 cm arms at 60°, 10 mm internal diameter). Purified air, passed sequentially through activated charcoal, distilled water and silica gel, was supplied at a constant flow rate of 0.5 L min^−1^ and delivered to each arm via Teflon tubing. Tomato plants corresponding to each treatment (CT, MCS and SCS) were individually enclosed in transparent plastic bags, with the upper ends connected to the airflow system to deliver plant volatiles into the olfactometer (Figure 2A). To minimize interference from soil-derived volatiles, pots were wrapped with aluminum foil. Individual adult females were collected directly from the corresponding temperature treatment groups described in Section 2.1 and tested immediately after collection. Each female was tested only once and was not reused in any subsequent assay. For each temperature treatment, 25 adult females (*n* = 25 per treatment) were used as independent biological replicates. Only females were selected because they are responsible for host plant selection and oviposition behavior in *T. absoluta*. Each individual was introduced at the base of the Y-tube and allowed to make a choice within a defined period (5 min). A response was recorded when an insect entered a choice arm, while individuals that did not make a definitive choice within the test period were excluded from the analysis, as they were not considered valid behavioral responses. For each assay, one arm contained plant volatiles and the other contained clean air as a control. Odor sources were alternated after every five individuals and the apparatus was cleaned with ethanol and dried between trials [28,31].

### 2.6. Metabolomic Profiling and Analysis

#### 2.6.1. Sample Preparation and LC-MS/MS Analysis

Untargeted metabolomic analysis was performed using *T. absoluta* individuals from the three temperature treatments (CT, MCS and SCS). Eight biological replicates (*n* = 8) were used for each temperature treatment. Frozen samples were thawed on ice and approximately 50–100 mg of *T. absoluta* tissue was transferred into centrifuge tubes, followed by the addition of 200 μL of pre-chilled water and vortexing for 60 s. Subsequently, 800 μL of cold methanol/acetonitrile (1:1, *v*/*v*) was added and samples were vortexed and subjected to low-temperature ultra-sonication for 30 min to facilitate metabolite extraction. The mixtures were then incubated at −20 °C for 1 h to precipitate proteins and centrifuged at 12,000 rpm for 10 min at 4 °C. The resulting supernatants were collected, vacuum-dried and reconstituted in 200 μL of 30% acetonitrile, followed by vortexing and centrifugation at 14,000 rpm for 15 min at 4 °C. Final extracts were transferred to auto-sampler vials for analysis. Quality control (QC) samples were prepared by pooling equal aliquots from all samples and were injected periodically to monitor analytical stability.

Metabolomic profiling was conducted using an ultra-performance liquid chromatography system coupled with high-resolution mass spectrometry (UPLC–HRMS). Chromatographic separation was achieved on a Waters HSS-T3 column (100 × 2.1 mm, 1.8 μm) at 40 °C, using a binary mobile phase consisting of water with 0.1% formic acid (A) and acetonitrile with 0.1% formic acid (B), delivered at a flow rate of 0.3 mL min^−1^ with an injection volume of 2 μL. A gradient elution program was applied, increasing from the aqueous phase to the organic phase and returning to initial conditions for equilibration. Samples were maintained at 4 °C and analyzed in randomized order. Mass spectrometric detection was performed using a Q-Exactive HFX mass spectrometer (Thermo Fisher Scientific, Waltham, MA, USA) equipped with an electrospray ionization (ESI) source operating in both positive and negative ion modes. Data were acquired in full MS-ddMS^2^ mode over an *m*/*z* range of 70–1050, with primary and secondary resolutions of 70,000 and 17,500, respectively. Ion source parameters included a sheath gas flow of 40 arb, auxiliary gas flow of 10 arb, spray voltage of +3000 V (positive) or −2800 V (negative), capillary temperature of 350 °C and ion transfer tube temperature of 320 °C [32,33].

#### 2.6.2. Data Processing and Metabolite Analysis

Raw LC–MS/MS data were processed using Progenesis QI software for peak detection, retention time alignment and normalization, generating a comprehensive data matrix of retention time, *m*/*z* and peak intensity. Metabolite identification was achieved by matching accurate mass and MS/MS fragmentation patterns against commercial databases and an in-house library and only confidently annotated metabolites were retained for further analysis. Multivariate analyses, including principal component analysis (PCA) and partial least squares discriminant analysis (PLS-DA), were applied to evaluate metabolic differences among treatments. Differential metabolites were identified based on statistical significance (adjusted *p* < 0.05) and ranked by absolute log2 fold change (|log2FC|) across pairwise comparisons and annotated metabolites were identified using the KEGG database [32].

### 2.7. Transcriptomic Profiling and Analysis

#### 2.7.1. RNA Isolation and Library Preparation

*T. absoluta* samples (4–6 individuals) were homogenized in TRIzol™ reagent (Invitrogen, Carlsbad, CA, USA) under RNase-free conditions and total RNA was extracted using a commercial kit (N066; Nanjing Jiancheng Bioengineering Institute, Nanjing, China) following the manufacturer’s instructions. RNA concentration and purity were assessed using a NanoDrop 2000 spectrophotometer (Thermo Fisher Scientific, Waltham, MA, USA), while integrity was verified by agarose gel electrophoresis and an Agilent 2100 Bioanalyzer (RIN ≥ 7.0). Sequencing libraries were prepared using the NEBNext^®^ Ultra™ RNA Library Prep Kit (New England Biolabs, Ipswich, MA, USA). Poly(A)+ mRNA was enriched using oligo(dT) beads, fragmented and reverse-transcribed into cDNA, followed by second-strand synthesis, adaptor ligation, PCR amplification and size selection. Four biological replicates (*n* = 4) were used for CT and MCS treatment while three biological replicates (*n* = 3) were used for SCS treatment.

#### 2.7.2. Sequencing, Differential Expression and Functional Analysis

Prepared libraries were sequenced on an Illumina platform to generate paired-end reads (2 × 150 bp). Raw reads were processed using fastp to remove adaptors, low-quality sequences and ambiguous reads and the resulting clean reads were aligned to the *T. absoluta* reference genome (GCA_027580185.1) using HISAT2. Sequencing quality was evaluated based on base quality scores, GC content and read length distribution. Gene expression levels were quantified from uniquely mapped reads and differential expression analysis was performed using DESeq2. Genes with an adjusted *p*-value < 0.05 and |log_2_ fold change| ≥ 1 were considered significantly differentially expressed, with expression levels additionally represented as FPKM values. Functional annotation and enrichment analyses were conducted using Gene Ontology (GO) and KEGG databases to identify significantly enriched biological processes, molecular functions, cellular components and metabolic pathways associated with temperature stress (adjusted *p*-value < 0.05).

### 2.8. Integrative Analysis

Differentially expressed genes (DEGs) were selected using a threshold of |log_2_ fold change| ≥ 1 and adjusted *p*-value (padj) ≤ 0.05. Similarly, significantly altered metabolites were identified based on an adjusted *p*-value ≤ 0.05. Pearson correlation analysis was performed to assess relationships between DEGs and significantly altered metabolites across all samples. Correlations with an absolute coefficient (|r|) > 0.85 and *p* < 0.01 were considered statistically significant and used for network construction. To visualize global coregulation patterns, a correlation heatmap was constructed using Pearson correlation coefficients between selected genes and metabolites. Weighted gene co-expression network analysis (WGCNA) was conducted to identify modules of co-expressed genes and module eigengenes were calculated and correlated with stress conditions to determine stress-associated modules. Furthermore, module–metabolite relationships were evaluated by correlating module eigengenes with metabolite abundance profiles. For visualization, a subset of metabolites with the highest variance and strongest statistical significance was selected to improve interpretability and reduce redundancy in high-dimensional data.

### 2.9. Statistical Analysis

Treatment effects were examined with analysis of variances (ANOVAs) using the “aov” function. When significant differences were observed (*p* < 0.05), pairwise mean comparisons were conducted using Tukey’s honest significant difference (HSD) test through the “TukeyHSD function”. Oviposition data, treated as count responses, were analyzed using a generalized linear model (GLM) with a Poisson error distribution via the “glm” function. Choice behavior data from the Y-tube olfactometer assay were evaluated with chi-square (*χ*^2^) tests to assess significant preferences between treatments controls. Data visualization and graphical outputs were generated using the ggplot2 package. All the statistical computations and graphics were performed using R (version 4.1.0; R Core Team, 2017).

## 3. Results

### 3.1. Low Temperature Impairs T. absoluta Adult Development and Physiological Performances

**Figure 1:**
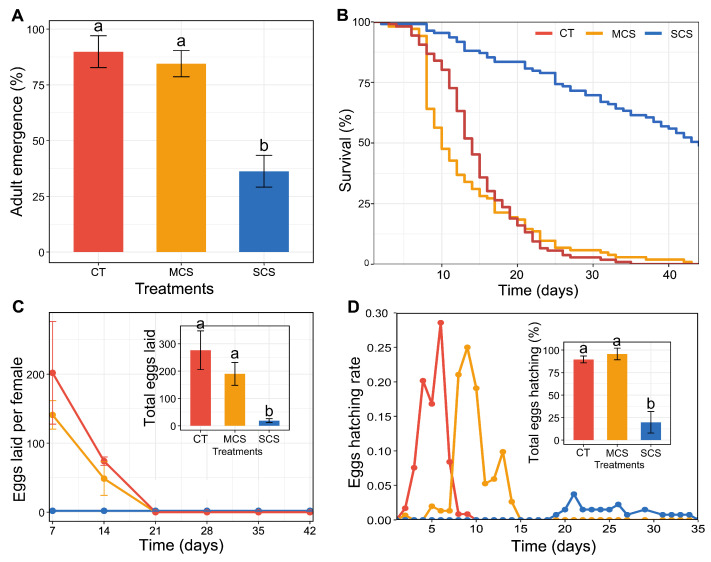
Effect of low temperature on *T. absoluta* adult emergence, survival, fecundity and egg hatching. (**A**) Adult emergence rate (*n* = 8 tests/treatment). (**B**) Survival of *T. absoluta* (*n* = 14 tests/treatment). (**C**) Daily egg laying dynamics over time (*n* = 3 tests/treatment). (**D**) Daily egg hatching over time across treatments (*n* = 3 tests/treatment). Bars represent means ± SE. Distinct letters above bars denote statistically significant differences (GLM, ANOVA *p* < 0.05).

Low temperature caused significant and negative effects to adult *T. absoluta* emergence, survival, reproductive output and host-seeking behavior across all measured parameters (Figure 1). Low temperature significantly reduced adult emergence rate (*F*_2, 21_ = 153.5, *p* < 0.000), with CT and MCS adults emerging at the highest rate and SCS causing the most severe suppression of adult eclosion, indicating that cold exposure during development compromises the successful completion of metamorphosis (Figure 1A). Adult survival trajectories showed time-dependent and treatment-dependent differences, where SCS adults maintained the highest survival across the observation period (Figure 1B). The reproductive consequences of low temperature were equally pronounced, as total eggs laid per female declined markedly with increasing stress intensity, with CT females producing the greatest egg output, MCS females showing a slight but not significant reduced fecundity and SCS females exhibiting near-complete reproductive suppression (Figure 1C; *F*_2, 6_ = 22.48, *p* < 0.001). In CT and MCS females, egg laying peaked at day 7 with the highest daily fecundity, followed by a sharp reduction by day 14 and near-complete cessation by day 21, while SCS females showed minimal egg laying throughout the observation period. This reproductive impairment extended beyond egg production, as egg hatching dynamics followed a temperature-dependent pattern, with CT eggs achieving the highest hatching success, MCS-derived eggs showing no significant difference compared to CT and SCS-derived eggs displaying the most severe reduction in hatching rate (Figure 1D; *F*_2, 6_ = 80.87, *p* < 0.000). Temporal analysis further revealed differences in hatching trajectories among treatments, with CT and MCS showing earlier and more pronounced peak hatching rates, whereas SCS exhibited delayed and strongly suppressed hatching over time, indicating altered developmental progression under low-temperature stress.

**Figure 2:**
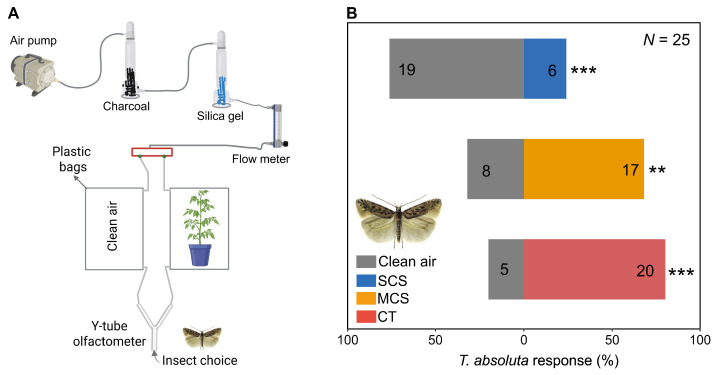
Olfactory response of low-temperature-stressed *T. absoluta* females to tomato plants. (**A**) Schematic illustration showing Y-tube olfactometer setup. (**B**) Olfactory response of *T. absoluta* females. Numbers within bars indicate the individuals making each choice. Significance levels: ** *p* < 0.01, *** *p* < 0.001 following *χ*^2^ test.

Furthermore, low temperature fundamentally altered adult female host plant orientation behavior, as CT adults exhibited a strong and significant preference for the tomato plant-scented arm over clean air in the Y-tube olfactometer (*χ*^2^ = 18.0, *p* < 0.000). MCS adults showed a reversed orientation pattern with reduced attraction toward the host plant (*χ*^2^ = 6.48, *p* < 0.010) and SCS adults displayed the most dramatic suppression of host-seeking behavior, showing the weakest plant preference (*χ*^2^ = 13.52, *p* < 0.000). These results indicate that low temperature progressively disrupts the olfactory-mediated host location capacity of adult *T. absoluta* (Figure 2A,B).

Taken together, these findings demonstrate that low temperature imposes a multi-dimensional physiological and behavioral cost on adult *T. absoluta*, simultaneously compromising emergence success, survivorship, reproductive capacity and host plant orientation.

### 3.2. Low Temperature Disrupts Energy Metabolism and Antioxidant Defense Systems

**Figure 3:**
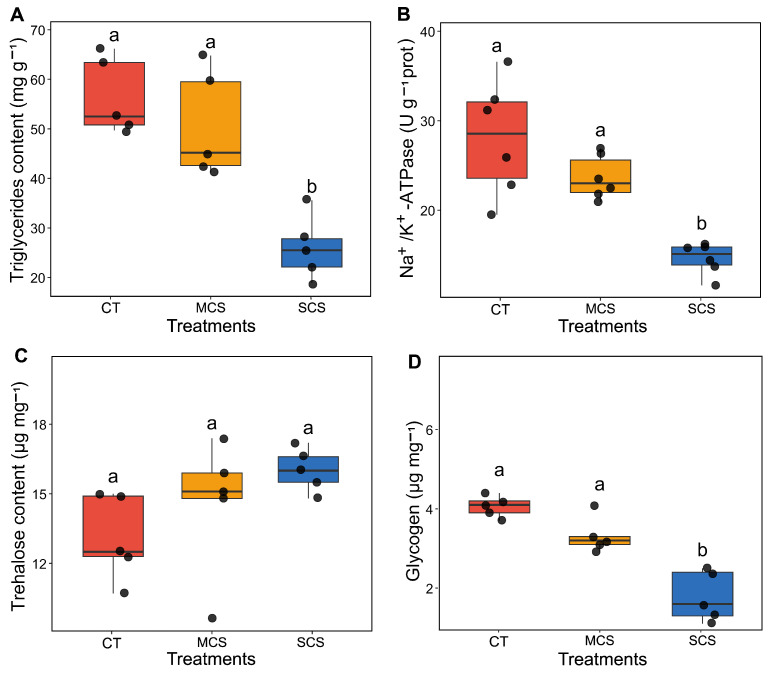
Effect of low temperature on energy metabolism in *T. absoluta*. (**A**) Triglyceride content (*n* = 5 tests/treatment). (**B**) Na^+^/K^+^-ATPase activity (*n* = 6 tests/treatment). (**C**) Trehalose content and (**D**) glycogen content across treatments (*n* = 5 tests/treatment). Black dots within bars represent individual data points. Distinct letters above bars denote statistically significant differences (ANOVA *p* < 0.05).

**Figure 4:**
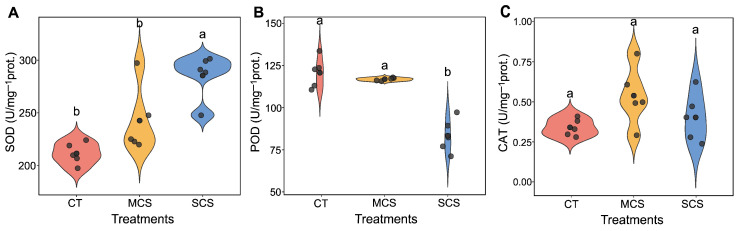
Effect of low temperature on antioxidant enzyme activities in *T. absoluta*. (**A**) SOD activity. (**B**) POD activity and (**C**) CAT activity across treatments (*n* = 5 tests/treatment). Violin plots display data distribution with individual data points overlaid. Black dots within bars represent individual data points. Distinct letters above bars denote statistically significant differences (ANOVA *p* < 0.05).

Low temperature significantly disrupted the energy metabolic homeostasis and antioxidant defense capacity of *T. absoluta* in a stress intensity-dependent manner (Figure 3 and Figure 4). Triglyceride content remained comparable between CT and MCS adults but declined sharply under SCS, with severe low temperature inducing the most pronounced depletion of lipid reserves (Figure 3A; *F*_2, 12_ = 18.38, *p* < 0.000), reflecting the substantial energetic cost imposed by extreme thermal challenge. Similarly, Na^+^/K^+^-ATPase activity, a critical indicator of membrane ion transport function, was maintained under MCS but significantly suppressed under SCS conditions compared to CT (Figure 3B; *F*_2, 15_ = 16.79, *p* < 0.000), suggesting that severe low temperature compromises membrane-associated bio-energetic processes essential for cellular homeostasis. In contrast, trehalose content showed a non-significant increasing trend with decreasing temperature, with SCS adults exhibiting higher levels compared to CT individuals (Figure 3C; *F*_2_, _12_ = 2.498, *p* < 0.124). Although not statistically significant, this pattern may suggest a potential metabolic adjustment under cold stress. Glycogen reserves followed a pattern parallel to triglycerides, remaining statistically comparable between CT and MCS adults while declining most severely under SCS treatment (Figure 3D; *F*_2, 12_ = 29.33, *p* < 0.000), collectively indicating that severe low temperature drives extensive mobilization and depletion of both lipid and carbohydrate energy stores in adult *T. absoluta*.

Furthermore, the antioxidant defense response to low temperature was equally complex and parameter-specific, as SOD activity was significantly elevated in SCS adults relative to CT, suggesting a biphasic enzymatic response wherein moderate low temperature activates superoxide scavenging capacity whereas severe stress may overwhelm or suppress this defense mechanism (Figure 4A; *F*_2, 12_ = 12.73, *p* < 0.001). POD activity declined under SCS conditions relative to both CT and MCS adults, indicating that severe low temperature impairs peroxidase mediated detoxification capacity (Figure 4B; *F*_2, 12_ = 32.81, *p* < 0.000), while CAT activity remained statistically unchanged across all three treatments, suggesting that catalase-mediated hydrogen peroxide decomposition is relatively resilient to thermal stress in this species (Figure 4C; *F*_2, 12_ = 2.521, *p* < 0.122).

### 3.3. Low Temperature Induces Comprehensive Metabolic Reprogramming in T. absoluta

#### 3.3.1. Metabolomic Profiling and Differential Metabolite Analysis

**Figure 5:**
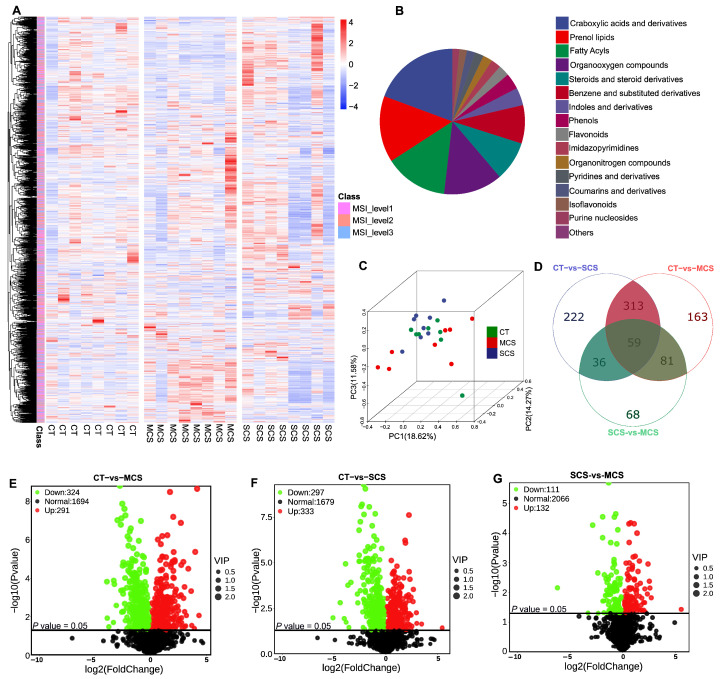
Untargeted metabolomic profiling and differential metabolite analysis of *T. absoluta* under low temperature. (**A**) Heatmap of normalized metabolite abundances across treatments. Class indicates metabolite annotation confidence levels based on MSI standards (MSI_level1–MSI_level3). (**B**) The chemical class distribution of all detected metabolites. (**C**) Principal component analysis showing distinct metabolic profiles across treatments. (**D**) Number of significantly altered metabolites. (**E**–**G**) Volcano plots displaying significantly upregulated (red) and downregulated (green) metabolites with variable importance in projection scores (*n* = 8 replicates/treatment). Black dots indicate non-significant genes.

To characterize the comprehensive metabolic consequences of low temperature in *T. absoluta* adults, we performed untargeted metabolomic profiling across all treatment (Figure 5). The metabolomic dataset revealed pronounced treatment-specific metabolic signatures, with hierarchical clustering of normalized metabolite abundances demonstrating clear segregation among CT, MCS and SCS adults, wherein cold-stressed samples displayed divergent metabolic profiles relative to control (Figure 5A). The chemical landscape of the detected metabolome was highly diverse, encompassing multiple structural classes with carboxylic acids and derivatives, prenol lipids, fatty acyls, organooxygen compounds, steroids and steroid derivatives, reflecting the broad biochemical scope of the low-temperature metabolic response (Figure 5B). The chemical composition assessed at the subclass level was dominated by lipids and lipid-like molecules while superclass level analysis further highlighted the diversity of metabolite categories (Figure S1A,B). PCA further confirmed the treatment-dependent metabolic restructuring, with CT, MCS and SCS adults forming clearly resolved clusters in PCA space, with PC1, PC2 and PC3 explaining 18.62%, 14.27% and 11.58% of total variance respectively (Figure 5C). Pairwise inter-sample correlation analysis revealed that CT adults maintained strong positive correlations within the treatment group, indicating highly consistent and reproducible metabolomic profiles under optimal thermal conditions, whereas MCS and SCS adults displayed progressively weakened within-group and between-group correlations, with SCS adults showing the most divergent metabolomic profiles relative to controls (Figure S2A–C).

Differential metabolite analysis across pairwise comparisons identified extensive and treatment-specific patterns of metabolic dysregulation (Figure 5D–G). Venn diagram analysis revealed that 163 metabolites were uniquely dysregulated in the CT-vs-MCS comparison, 222 metabolites were unique to the CT-vs-SCS comparison and 68 metabolites were unique to the SCS-vs-MCS comparison (Figure 5D). Notably, 313 metabolites were commonly dysregulated across both CT-vs-MCS and CT-vs-SCS comparisons, while 59 metabolites were shared across all three pairwise comparisons, representing a conserved low-temperature metabolic signature in *T. absoluta*. Individual comparisons revealed that the CT-vs-MCS comparison identified 324 downregulated and 291 upregulated metabolites (Figure 5E), while the CT-vs-SCS comparison yielded 297 downregulated and 333 upregulated metabolites, representing the most extensive metabolic divergence across all comparisons (Figure 5F). The SCS-vs-MCS comparison identified 111 downregulated and 132 upregulated metabolites, reflecting the incremental but significant metabolic shift between the two low-temperature intensities (Figure 5G). Furthermore, dynamic trajectory analysis of metabolite subclasses across the stress gradient revealed divergent and coordinated expression patterns, wherein subclass 5, comprising the largest cohort of 771 metabolites, showed progressive suppression from SCS to CT conditions (Figure S3). These comprehensive metabolomic profiling results demonstrate that low temperature triggers systematic and progressive metabolic reprogramming in *T. absoluta*.

#### 3.3.2. Pathway Enrichment Analysis and Key Metabolite Alterations

**Figure 6:**
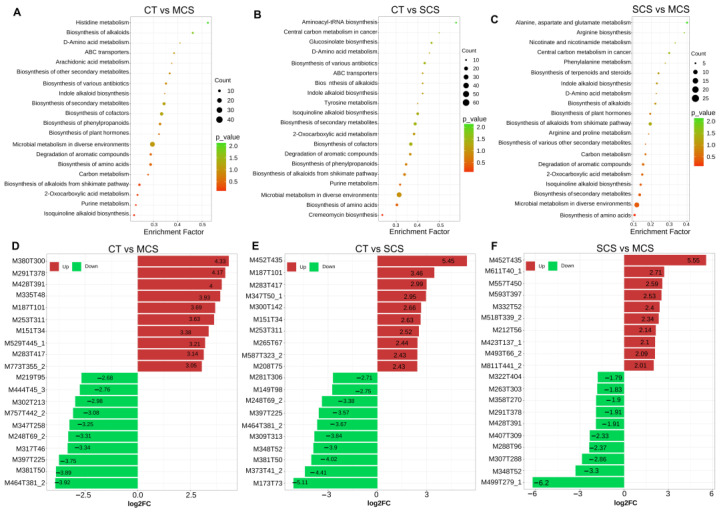
KEGG pathway enrichment analysis and key differentially accumulated metabolites in *T. absoluta* under low temperature. (**A**–**C**) KEGG plot showing enriched pathways, with bubble size representing the number of detected metabolites and color intensity indicating *p*-value significance. (**D**–**F**) Log_2_ fold change bar plot of dysregulated metabolites, showing individual metabolite identifiers with upregulated (red) and downregulated (green) metabolites.

Pathway enrichment analysis revealed that low temperature systematically targets core and specialized metabolic pathways in *T. absoluta* adult in a stress intensity-dependent manner (Figure 6). In the CT-vs-MCS comparison, the most prominently enriched pathways included histidine metabolism, biosynthesis of alkaloids, D-amino acid metabolism, ABC transporters and biosynthesis of cofactors, indicating that even moderate low temperature triggers broad metabolic reorientation spanning both primary and specialized biosynthetic routes (Figure 6A). The CT-vs-SCS comparison revealed a more extensive and severe pattern of pathway dysregulation, with aminoacyl-tRNA biosynthesis, central carbon metabolism, glucosinolate biosynthesis, tyrosine metabolism, isoquinoline alkaloid biosynthesis, biosynthesis of phenylpropanoids, and purine metabolism among the most significantly enriched pathways, indicating that severe low temperature induces a fundamental restructuring of both translational machinery and secondary metabolic networks in *T. absoluta* (Figure 6B). The SCS-vs-MCS comparison further identified alanine, aspartate and glutamate metabolism, arginine biosynthesis, nicotinate and nicotinamide metabolism, phenylalanine metabolism, biosynthesis of terpenoids and steroids, indole alkaloid biosynthesis and carbon metabolism as significantly enriched pathways (Figure 6C).

Individual metabolite analysis across pairwise comparisons revealed consistent and biologically meaningful patterns of dysregulation at the metabolite level (Figure 6D–F). In the CT-vs-MCS comparison, the most strongly upregulated metabolites included M380T300, M291T378, M428T391 and M335T48, while the most severely downregulated metabolites included M464T381_2, M381T50 and M397T225, indicating substantial bidirectional metabolic shifts even under MCS conditions (Figure 6D). The CT-vs-SCS comparison yielded the most extreme individual metabolite fold changes across all comparisons, with M452T435 showing the highest upregulation and M173T73 displaying the most severe downregulation, reflecting the profound metabolic disruption imposed by SCS (Figure 6E). Notably, several metabolites including M187T101, M253T311, M151T34, M248T69_2, M397T225, M464T381_2 and M381T50 appeared as consistently dysregulated across both CT-vs-MCS and CT-vs-SCS comparisons. In the SCS-vs-MCS comparison, M452T435 again emerged as the most strongly upregulated metabolite, while M499T279_1 showed the most dramatic downregulation, confirming the stress intensity-dependent nature of metabolite accumulation and depletion patterns (Figure 6F). These pathway enrichment and individual metabolite analyses demonstrate that low temperature drives comprehensive and progressive metabolic reprogramming in *T. absoluta* adults.

### 3.4. Low-Temperature Treatment Triggers Coordinated Gene Expression Reprogramming in T. absoluta

#### 3.4.1. Global Gene Expression Profiling and Differential Expression Analysis

**Figure 7:**
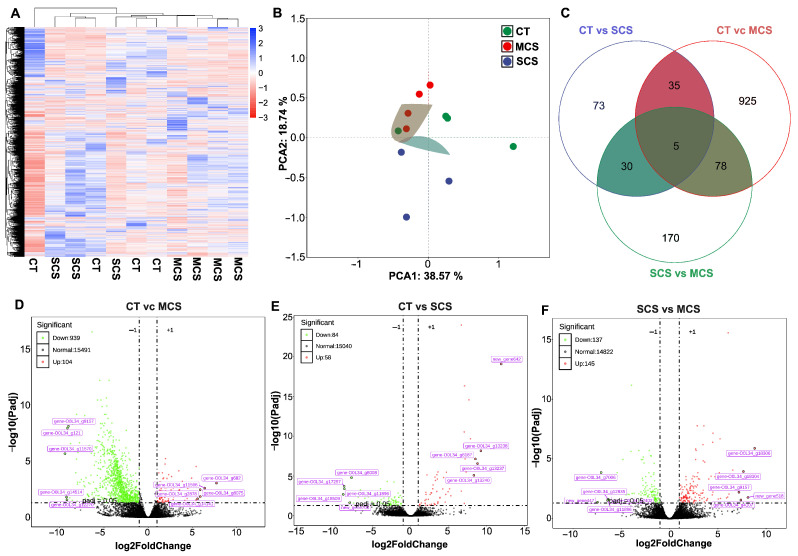
Global transcriptomic profiling and differential gene expression analysis of *T. absoluta* under low temperature. (**A**) Heatmap of normalized gene expression levels across treatments. (**B**) Principal component analysis score plot showing treatment group separation. (**C**) Unique and shared differentially expressed genes (DEGs) across comparisons. (**D**–**F**) Volcano plots of DEGs; green dots indicate downregulated, red dots indicate upregulated and black dots indicate non-significant genes; dashed lines indicate padj = 0.05 and log2FoldChange thresholds of ±1. CT, MCS; (*n* = 4 replicates/each), SCS (*n* = 3 replicates).

To comprehensively characterize the transcriptional consequences of low-temperature exposure in *T. absoluta* adults, we performed RNA sequencing and analyzed global gene expression patterns (Figure 7). The transcriptomic dataset revealed pronounced treatment-specific transcriptional signatures, with hierarchical clustering of normalized gene expression levels demonstrating clear segregation among CT, MCS and SCS treatments, wherein cold-stressed samples displayed progressively divergent transcriptomic profiles relative to the control (Figure 7A). PCA confirmed this treatment-dependent transcriptional divergence, with CT, MCS and SCS adults forming clearly resolved clusters in reduced dimensional space, wherein PC1 and PC2 collectively explained 38.57% and 18.74% of total variance respectively and SCS adults exhibited the greatest displacement from control conditions (Figure 7B). The analysis of pairwise DEG comparisons revealed distinct patterns of unique and shared transcriptional responses across low-temperature intensities (Figure 7C). The CT-vs-MCS comparison yielded the largest number of uniquely dysregulated genes (925), while CT-vs-SCS and SCS-vs-MCS comparisons identified 73 and 170 unique DEGs respectively, indicating that MCS triggers a broader unique transcriptional response in *T. absoluta* than SCS alone. Notably, 35 genes were commonly dysregulated across CT-vs-SCS and CT-vs-MCS comparisons, while five genes were shared across all three pairwise comparisons, indicating a conserved transcriptional low-temperature signature in *T. absoluta*. The presence of 78 genes co-dysregulated in both CT-vs-MCS and SCS-vs-MCS comparisons and 30 genes shared between CT-vs-SCS and SCS-vs-MCS comparisons further highlights the hierarchical and temperature-dependent nature of transcriptional stress pathway activation in this species.

Furthermore, pairwise comparisons identified extensive transcriptional dysregulation across all treatment contrasts. The CT-vs-MCS comparison revealed the most substantial gene expression alterations, with 939 downregulated and 104 upregulated genes identified, indicating that MCS predominantly suppresses gene expression in *T. absoluta* (Figure 7D). The CT-vs-SCS comparison showed comparatively fewer but highly significant expression changes, with 84 downregulated and 58 upregulated genes, reflecting a more selective and targeted transcriptional response under extreme low temperature (Figure 7E). The SCS-vs-MCS comparison identified 137 downregulated and 145 upregulated genes, demonstrating bidirectional and incremental transcriptional reorientation as low-temperature intensity transitions from moderate to severe (Figure 7F). These results demonstrate that low temperature induces progressive and treatment-specific transcriptional reprogramming in *T. absoluta* adults, with the nature and magnitude of gene expression changes varying systematically with thermal stress intensities.

#### 3.4.2. Functional Pathway Enrichment and Transcriptional Reprogramming

**Figure 8:**
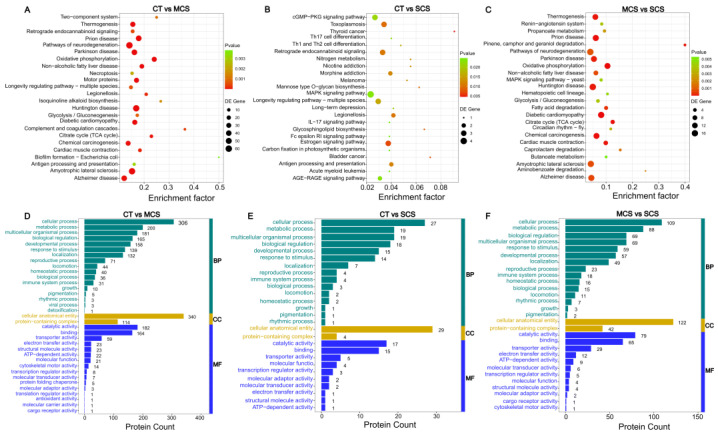
KEGG pathway enrichment and Gene Ontology analysis of differentially expressed genes in *T. absoluta* under low temperature. (**A**–**C**) KEGG enrichment; dot size represents the number of differentially expressed genes enriched in each pathway and color indicates the *p*-value. (**D**–**F**) GO bar charts showing protein counts across biological process, cellular component and molecular function categories.

Pathway enrichment analysis using KEGG databases revealed that low temperature systematically remodels core metabolic, neurological and stress response pathways in *T. absoluta* adults (Figure 8). In the CT-vs-MCS comparison, thermogenesis, retrograde endocannabinoid signaling, prion disease, pathways of neurodegeneration, Parkinson disease, oxidative phosphorylation and cardiac muscle contraction emerged as significantly enriched pathways, indicating that MCS triggers broad metabolic reorientation spanning energy metabolism, neurodegeneration signaling and immune-related processes (Figure 8A). The CT-vs-SCS comparison revealed a distinctly different and complementary pattern of pathway dysregulation, with cGMP-PKG signaling, MAPK signaling, retrograde endocannabinoid signaling, Th17 and Th1/Th2 cell differentiation, nitrogen metabolism, nicotine and morphine addiction pathways, mannose-type O-glycan biosynthesis, the longevity regulating pathway and antigen processing reflecting a fundamental shift toward signal transduction, immune modulation and glycan remodeling under SCS (Figure 8B). The MCS-vs-SCS comparison identified thermogenesis, the renin–angiotensin system, propanoate metabolism, prion disease, pathways of neurodegeneration, Parkinson’s disease, oxidative phosphorylation, MAPK signaling, Huntington’s disease, glycolysis and gluconeogenesis and Alzheimer’s disease as significantly enriched pathways, demonstrating that the transcriptional transition from MCS to SCS drives additional activation of energy catabolism, circadian disruption and progressive neurodegeneration-associated pathway remodeling (Figure 8C). These functional classifications confirmed that neurodegenerative disease pathways constituted the most extensively represented category across all comparisons, with signal transduction, carbohydrate metabolism, energy metabolism and organismal system pathways consistently among the major functional domains affected by low temperature in *T. absoluta* (Figure S4A–C).

Gene Ontology analysis further characterized the functional scope of low-temperature-induced transcriptional changes across all three biological dimensions (Figure 8D–F). In the CT-vs-MCS comparison, biological process categories were dominated by cellular process, metabolic process, multicellular organismal process, biological regulation, developmental process and response to stimulus, with reproductive process and locomotion also substantially represented, indicating that MCS broadly disrupts fundamental biological and developmental processes in *T. absoluta* (Figure 8D). Cellular component analysis identified cellular anatomical entity and protein-containing complex as the primary structural targets, while molecular function categories highlighted binding and catalytic activity as the most extensively altered functional classes, alongside transporter activity and electron transfer activity. The CT-vs-SCS comparison yielded comparatively fewer but functionally significant GO enrichments, with cellular process, metabolic process, multicellular organismal process and biological regulation representing the dominant biological process categories and catalytic activity and binding as the primary molecular functions altered under severe low temperature (Figure 8E). The MCS-vs-SCS comparison exhibited intermediate GO enrichment, with cellular process, metabolic process, biological regulation, multicellular organismal process and response to stimulus as major biological process categories and catalytic activity and binding as the predominant molecular functions, confirming the progressive and dose-dependent nature of transcriptional reprogramming across low-temperature intensities (Figure 8F). Inter-sample correlation analysis demonstrated that CT samples maintained moderate within-group correlations, while MCS samples displayed higher within-group consistency and cross-treatment correlations were progressively reduced with increasing thermal divergence, with CT-SCS sample pairs showing the weakest correlations, confirming treatment-dependent transcriptional reorganization (Figure S5). These comprehensive transcriptomic analyses demonstrate that low temperature triggers systematic and progressive gene expression reprogramming in *T. absoluta* adults, simultaneously disrupting energy metabolism, neurodegeneration-associated signaling, immune response pathways and fundamental developmental processes, with the coordinated suppression of growth and reproductive functions.

### 3.5. Integrative Transcriptome–Metabolome Analysis Reveals Coordinated Molecular Networks Underlying Low-Temperature Responses in T. absoluta

**Figure 9:**
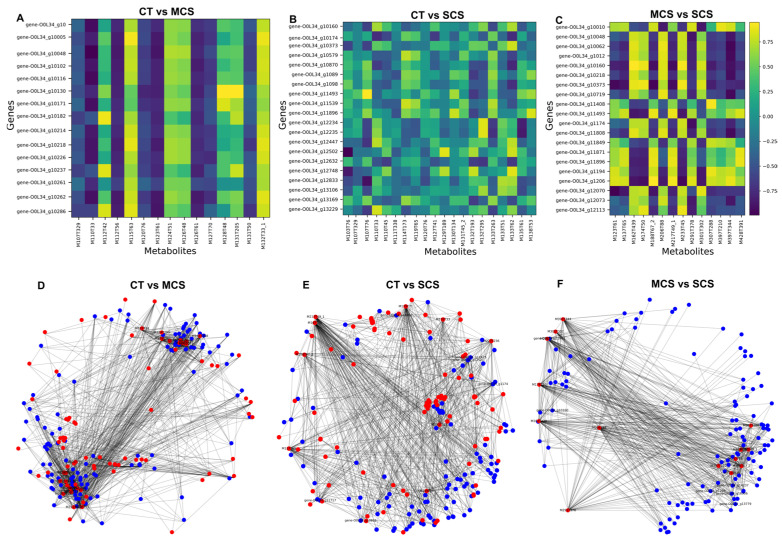
Integrative transcriptome–metabolome correlation analysis and gene–metabolite interaction networks in *T. absoluta* under low temperature. (**A**–**C**) Heatmaps displaying Pearson correlation coefficients between differentially expressed genes and differentially accumulated metabolites across comparisons; color scale ranges from strong negative (purple) to strong positive (yellow) correlations. (**D**–**F**) Gene–metabolite co-expression interaction networks; red nodes represent upregulated genes, blue nodes represent metabolites and edges indicate significant correlations between connected nodes.

To delineate the molecular regulatory architecture underlying low-temperature responses in *T. absoluta*, we performed integrative transcriptome–metabolome correlation analysis and WGCNA across all pairwise treatment comparisons (Figure 9 and Figure S6). Gene–metabolite correlation heatmap analysis revealed complex and treatment-specific association patterns between differentially expressed genes and differentially accumulated metabolites across all comparisons (Figure 9A–C). In the CT-vs-MCS comparison, gene–metabolite correlations displayed a heterogeneous pattern of both strong positive and negative associations (Figure 9A). The CT-vs-SCS comparison revealed more structured and pronounced correlation blocks, wherein specific gene clusters exhibited coherent positive or negative associations with defined metabolite groups (Figure 9B). The MCS-vs-SCS comparison displayed the most clearly defined alternating positive and negative correlation blocks, suggesting that the transcriptional transition between moderate and severe low temperature involves highly coordinated and opposing regulation of gene–metabolite relationships within specific biochemical domains (Figure 9C).

Gene–metabolite interaction network analysis further revealed the topology and complexity of low-temperature-induced molecular coregulation across treatment comparisons (Figure 9D–F). The CT-vs-MCS interaction network exhibited the densest connectivity, with numerous hub genes forming extensive interaction clusters with multiple metabolite nodes (Figure 9D). The CT-vs-SCS network showed a comparatively more focused architecture, with clearly identifiable hub genes maintaining high connectivity to defined metabolite clusters (Figure 9E). The MCS-vs-SCS network displayed the sparsest overall connectivity with distinct sub-clusters of blue nodes predominating, consistent with the incremental but biologically meaningful molecular divergence between the two low-temperature intensities (Figure 9F). WGCNA identified four co-expression modules (Module_0 through Module_3) across all three pairwise comparisons, each displaying distinct and contrasting metabolite correlation signatures (Figure S6). Module_1 and Module_2 exhibited the most pronounced and opposing module–metabolite correlation patterns across all comparisons, with Module_1 showing strong positive associations with specific metabolite subsets while Module_2 displayed correspondingly strong negative correlations with the same metabolites, suggesting that these modules represent functionally antagonistic transcriptional programs coordinately regulating complementary aspects of the low-temperature metabolic response. Collectively, these integrative analyses demonstrate that low temperature orchestrates a highly coordinated molecular response in *T. absoluta* adults through the concurrent reprogramming of gene expression and metabolite accumulation, with the complexity and specificity of gene–metabolite regulatory networks.

## 4. Discussion

Understanding how insect pests respond to thermal stress at physiological, biochemical and molecular levels is fundamental for predicting population dynamics under climate variability and for developing thermally informed pest management strategies [34,35]. *T. absoluta*, among the most economically destructive lepidopteran pests globally, has demonstrated remarkable capacity to colonize diverse climatic regions, yet the comprehensive physiological and molecular mechanisms supporting adult cold tolerance remain poorly understood [2,5]. This study addresses this knowledge gap by integrating biological performance assessments, biochemical analyses, metabolomics and transcriptomics to reveal the coordinated system-level responses of adult *T. absoluta* to thermal stress.

Low temperature imposes fundamental constraints on moth performance that extend beyond simple metabolic depression, forcing physiological trade-offs between survival, reproduction and stress tolerance [12]. Our results demonstrate that low temperature significantly suppressed adult emergence rate only under severe cold stress (SCS), while CT and MCS showed comparable eclosion success, indicating that cold exposure during development under severe conditions is required to impair successful metamorphic completion and adult viability. These emergence effects were accompanied by changed mortality trajectories, wherein CT or MCS adults displayed the most rapid survival decline than SCS, likely reflecting differential metabolic activity under varying thermal conditions, with severe cold potentially inducing metabolic suppression that delays mortality during the observation period [27]. The reproductive consequences of low temperature were equally profound, as fecundity and egg hatching rates declined systematically with increasing stress intensity, suggesting that low temperature simultaneously impairs maternal reproductive investment and offspring developmental viability [27,36]. Such reproductive suppression under thermal stress likely reflects the diversion of metabolic resources away from reproduction toward stress tolerance mechanisms, a fundamental life history trade-off documented across diverse insect taxa under environmental stress conditions [37,38,39,40]. Furthermore, low temperature fundamentally disrupted female host-seeking behavior, as demonstrated by the Y-tube olfactometer, wherein CT adults exhibited strong attraction toward tomato plant volatiles while MCS and SCS adults showed progressively reversed orientation responses with markedly reduced host plant preference. This low-temperature-induced disruption of olfactory perception has important ecological implications, as environmental temperature has been known to cause detectable olfactory perception changes in *Drosophila melanogaster* [41,42].

The physiological impairments observed in adult performance are mechanistically linked to pronounced disruptions in energy metabolic homeostasis under low temperature [35,43]. SCS resulted in substantial depletion of triglyceride and glycogen reserves, whereas MCS maintained energy levels comparable to controls, indicating that only extreme cold exceeds the buffering capacity of endogenous energy stores. This depletion likely reflects increased energetic demands for maintenance under stress coupled with reduced metabolic efficiency in ectothermic insects [44,45]. Consistently, Na^+^/K^+^-ATPase activity was suppressed under SCS, suggesting impaired ion transport and loss of cellular bio-energetic stability [46,47]. In contrast, trehalose content showed a non-significant increasing trend with stress intensity, reaching the highest levels under SCS, which may indicate a potential compensatory metabolic adjustment under low-temperature conditions in *T. absoluta* [48]. Together, these responses indicate a coordinated metabolic shift in *T. absoluta*, whereby energy reserves are mobilized and redirected from storage toward protective metabolite synthesis, prioritizing survival over growth and reproduction.

Concurrently, low temperature induced a complex and enzyme-specific antioxidant response, reflecting the generation of oxidative stress under low-temperature conditions [49,50]. SOD activity increased significantly under SCS, indicating enhanced superoxide scavenging capacity, whereas POD activity declined, suggesting impaired peroxidase-mediated detoxification under severe stress. In contrast, CAT activity remained relatively stable, implying greater thermal resilience of catalase-mediated hydrogen peroxide detoxification. These differential enzyme responses likely arise from variations in biochemical properties, subcellular localization and regulatory control and collectively suggest that low temperature disrupts redox homeostasis through multiple pathways, including mitochondrial dysfunction, reduced enzymatic efficiency, and direct cellular damage [51,52,53].

The physiological and biochemical alterations observed in adult *T. absoluta* under low temperature are further supported by coordinated metabolomic reprogramming [14,54,55]. Multivariate analyses demonstrated clear and progressive metabolic divergence with increasing stress intensity, indicating a systematic rather than random response. Low temperature broadly affected both primary and secondary metabolism, with significant alterations in lipid metabolism, amino acid pathways and central carbon metabolism, reflecting a shift in substrate utilization [14]. Differential metabolite patterns suggest concurrent suppression of energy-intensive processes alongside the selective accumulation of protective and stress-associated metabolites. Enrichment of pathways related to amino acid metabolism, the citrate cycle and secondary metabolite biosynthesis further indicates that low temperature reconfigures metabolic flux toward maintenance and survival [56,57]. These metabolic shifts demonstrate that adult low-temperature responses involve an organized metabolic restructuring that prioritizes cellular protection and stress adaptation over growth and biosynthetic activities.

The metabolomic shifts observed under low temperature are further supported by coordinated transcriptional reprogramming, indicating a mechanistic link between gene regulation and metabolic adaptations [57,58]. Cold exposure induced distinct, stress intensity-dependent transcriptional profiles, with moderate stress largely suppressing gene expression, whereas severe stress triggered a more balanced reorganization of up- and downregulated genes [20]. Pathway enrichment analysis revealed that low temperature primarily targets energy metabolism, including glycolysis, the citrate cycle and oxidative phosphorylation, alongside key signaling pathways such as MAPK, highlighting a broad disruption of cellular energy production and regulatory networks. Notably, the enrichment of neurodegeneration-associated pathways suggests impairment of mitochondrial function and protein homeostasis, reflecting conserved stress response mechanisms rather than disease-specific processes [59,60,61]. Gene Ontology patterns further indicate that low temperature acts as a system-level regulator, affecting metabolic processes, cellular organization and catalytic activity, thereby driving a comprehensive transcriptional shift that supports survival and stress adaptation [20,61].

Furthermore, the integrative transcriptomic–metabolomic analysis revealed a coordinated molecular response linking genes regulation to metabolic adaptations under low temperature in adult *T. absoluta* [14,62]. Distinct stress-dependent interaction patterns indicate that moderate cold induces broad molecular adjustments, whereas severe stress triggers more focused and targeted regulatory responses. The identification of co-expression modules with contrasting metabolite associations suggests the presence of functionally complementary transcriptional programs that differentially regulate energy metabolism and stress-protective pathways [63,64]. These analyses indicate that transcriptional reprogramming and metabolic shifts are tightly coupled rather than independent processes, collectively driving a unified adaptive response in which gene regulatory networks direct metabolic reorientation toward cellular protection and cold tolerance.

## 5. Conclusions

This study demonstrates that *T. absoluta* adults mount a coordinated and multi-layered response to low temperature, integrating physiological, metabolic and molecular adaptations into unified survival strategies. Rather than acting through isolated mechanisms, low temperature triggers an integrated regulatory network in which transcriptional control drives shifts in energy metabolism, cryoprotectant accumulation and antioxidant defenses, while concurrently suppressing reproduction, host-seeking behavior and growth. This coordinated prioritization of survival over reproductive investment highlights a fundamental adaptive strategy under thermal stress. These findings provide mechanistic insight into how a globally important pest responds to low-temperature challenges across multiple biological scales, from gene regulation to organismal performance, and underscore the importance of incorporating thermal stress biology into predictive models of pest dynamics and climate-resilient management strategies.

## Data Availability

The original contributions presented in this study are included in the article and Supplementary Materials. No additional datasets were generated or analyzed beyond those presented in the manuscript, Supplementary Materials and submitted supporting files. Further inquiries can be directed to the corresponding author.

## Supplementary Materials

The following supporting information can be downloaded at https://www.mdpi.com/article/10.3390/insects17070706/s1: Figure S1. Comprehensive classification of detected metabolites by chemical structure and metabolic origin; Figure S2. Pairwise metabolite correlation analysis under low temperature; Figure S3. Dynamic expression patterns of metabolite subclasses across temperature treatments; Figure S4. Comprehensive KEGG pathway enrichment analysis; Figure S5. Inter-sample pearson correlation heatmap of *T. absoluta* transcriptomes across low-temperature treatments; Figure S6. WGCNA module–metabolite correlation heatmaps in *T. absoluta* under low temperature.

## Abbreviations

The following abbreviations are used in this manuscript:

CT	Control stress
MCS	Moderate cold stress
SCS	Severe cold stress
DEGs	Differentially expressed genes
