# Supplementary material for: Low-Temperature Stress Impairs Reproductive Performance and Olfactory Behaviors in Tuta absoluta via Metabolic and Transcriptional Changes
Source: Insects. 2026 Jul 7;17(7):706. doi: 10.3390/insects17070706 (PMC13409891; doi:10.3390/insects17070706)
Supplement: Supplementary file 1 [file insects-17-00706-s001.zip › insects-4361222-supplementary.pdf]

Supporting Information

Low Temperature Stress Impairs Reproductive Performance and Olfactory Behaviors in *Tuta Absoluta* Via Metabolic and Transcriptional Changes

The following Supporting Information is available for this article:

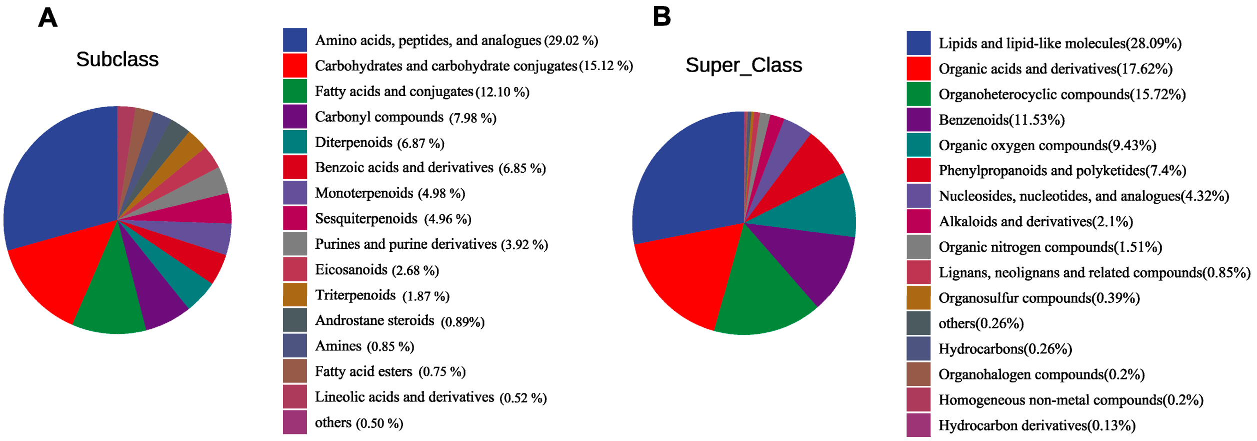

Fig. S1. Comprehensive classification of detected metabolites by chemical structure and metabolic origin. (**A**) Distribution of metabolites by subclass. (**B**) Super class categorization.

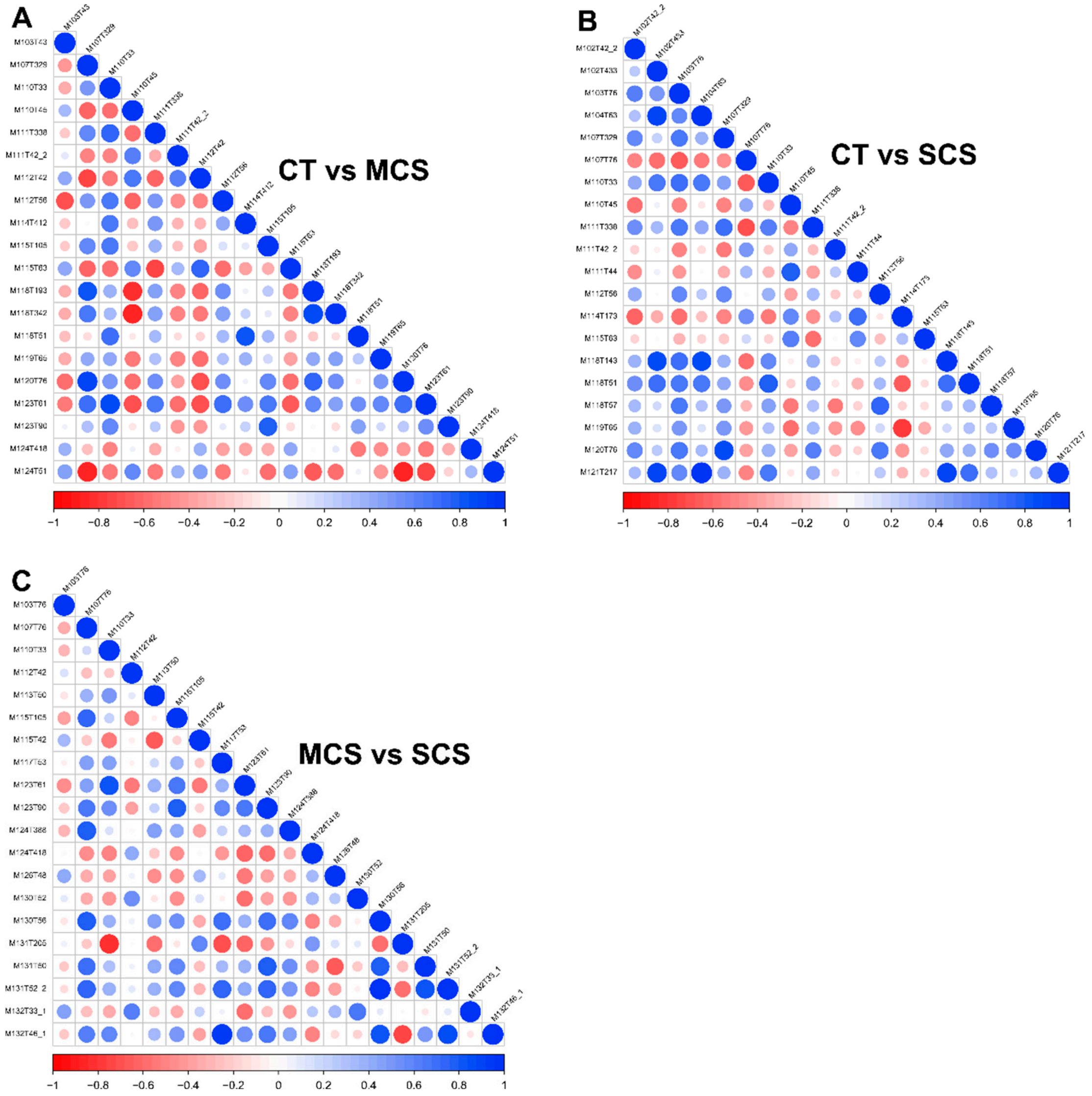

Fig. S2. Pairwise metabolite correlation analysis under low temperature. Correlation matrices for (**A**) CT vs MCS. (**B**) CT vs SCS and (**C**) MCS vs SCS comparisons; circle size and color intensity reflect the magnitude and direction of Pearson correlation coefficients.

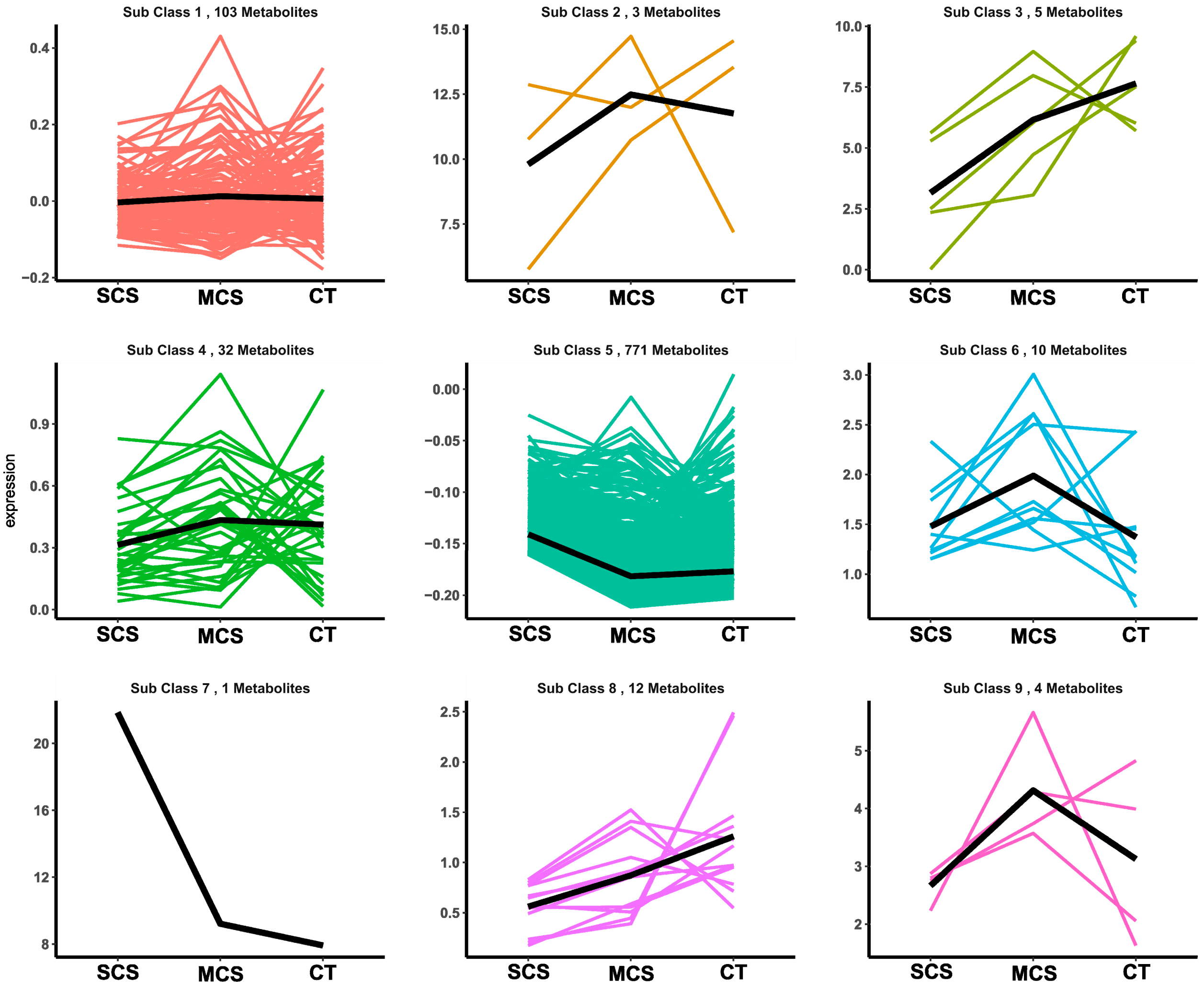

Fig. S3. Dynamic expression patterns of metabolite subclasses across temperature treatments. Each subplot represents a distinct metabolite subclass with individual metabolites shown as separate lines and the bold black line indicating the mean trajectory.

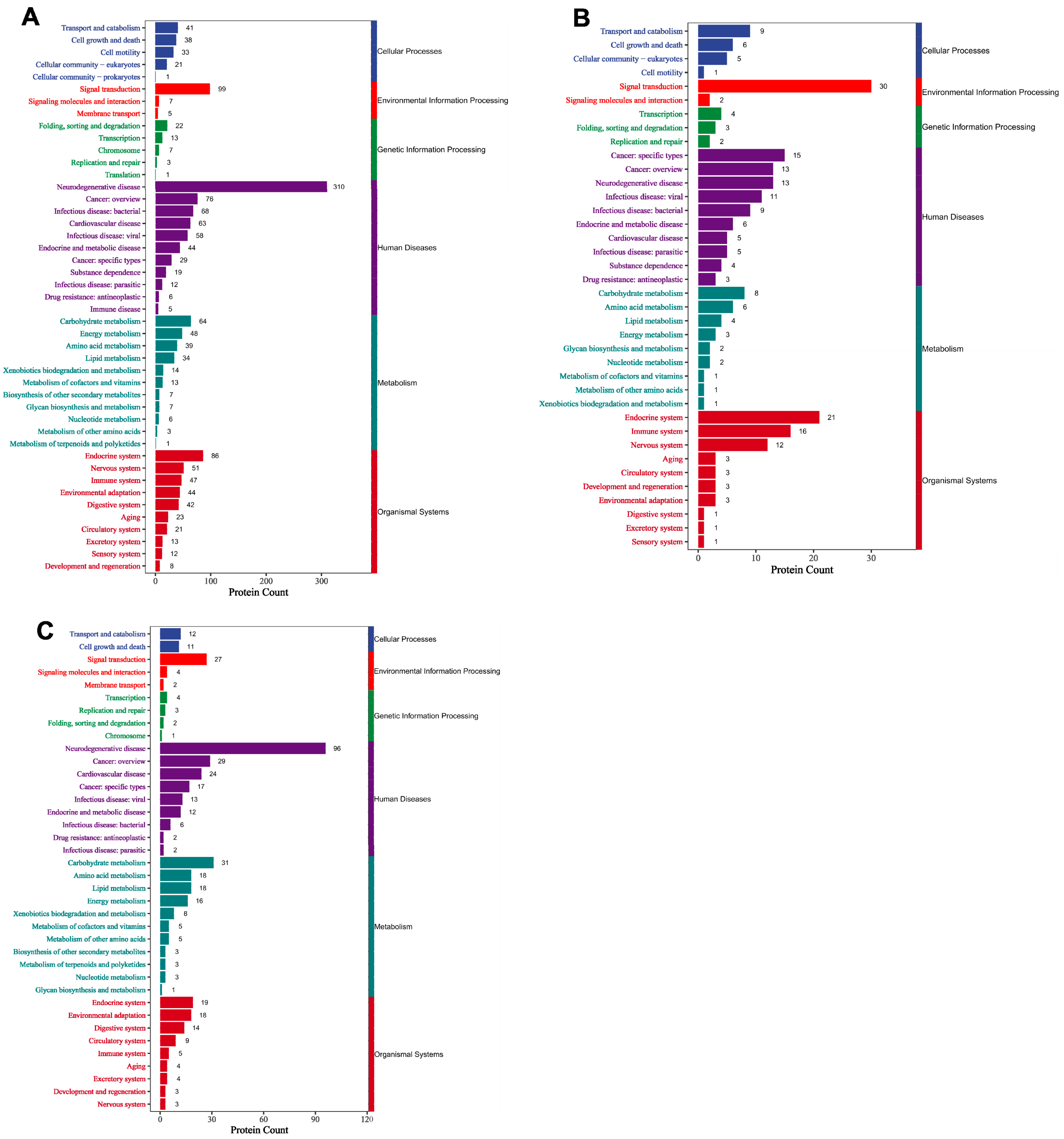

Fig. S4. (**A-C**) Comprehensive KEGG pathway enrichment analysis highlighting cellular processes, environmental information processing, genetic information processing, metabolism, human diseases and organismal systems categories across all treatment comparisons. (**A**) CT vs MCS. (**B**) CT vs SCS and (**C**) MCS vs SCS.

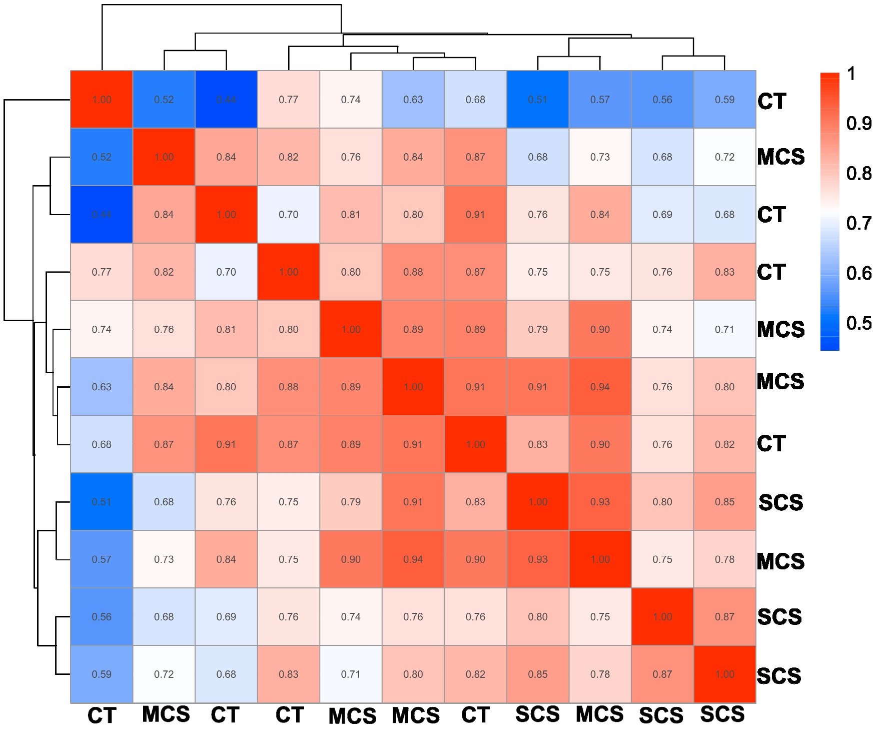

Fig. S5. Inter-sample pearson correlation heatmap of *T. absoluta* transcriptomes across low temperature treatments. Hierarchical clustering heatmap displaying pairwise correlation coefficients among all CT, MCS and SCS samples; color intensity reflects correlation magnitude from low (blue) to high (red).

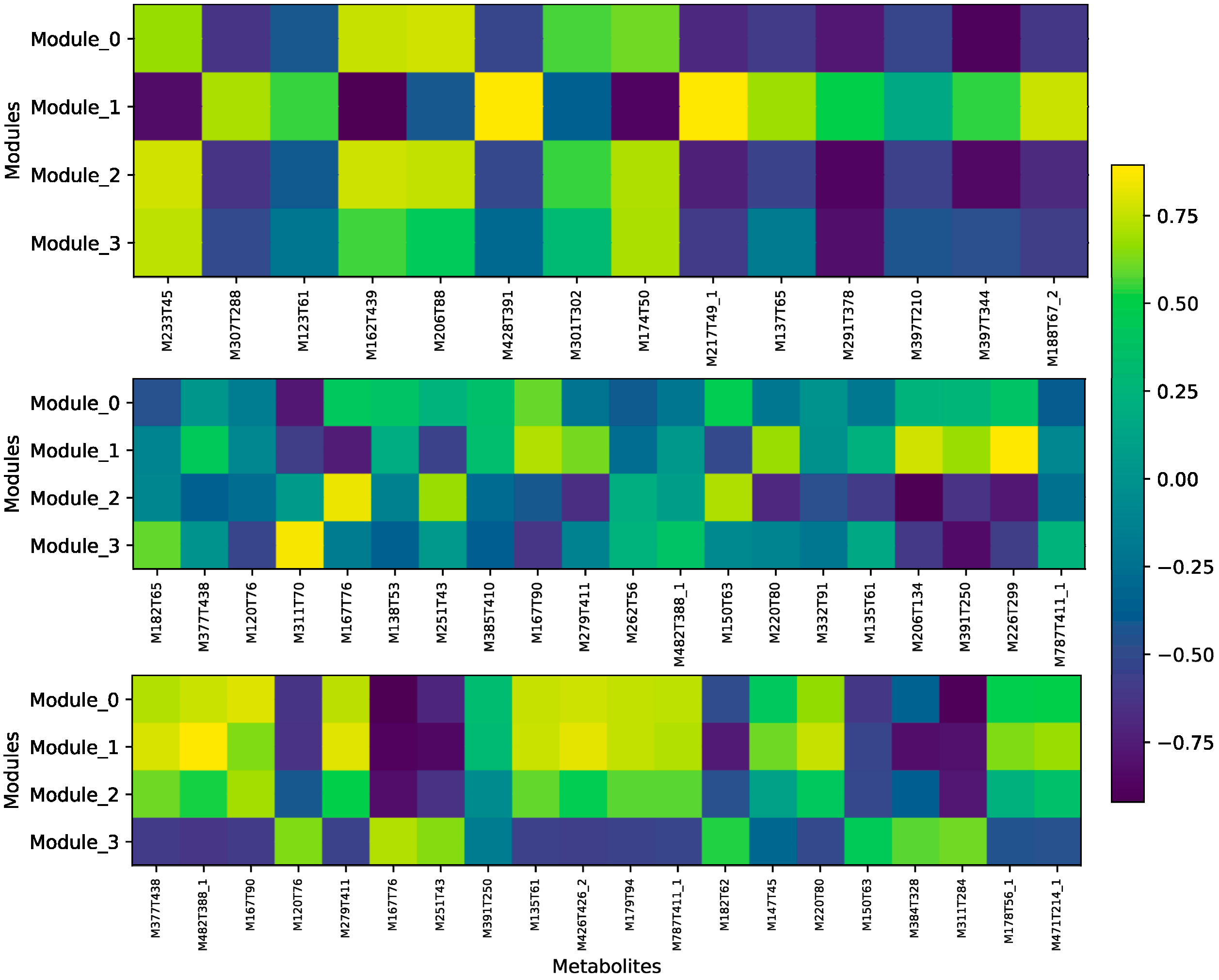

Fig. S6. WGCNA module-metabolite correlation heatmaps in *T. absoluta* under low temperature. Heatmaps showing correlation coefficients between WGCNA co-expression modules (Module_0 to Module_3) and key metabolites across CT-vs-MCS (top), CT-vs-SCS (middle) and MCS-vs-SCS (bottom) comparisons; color scale ranges from strong negative (purple) to strong positive (yellow) associations.
